# Frequency of non-alcoholic fatty liver disease in patients with type-2 diabetes mellitus and its associated risk factors

**DOI:** 10.12669/pjms.37.5.4211

**Published:** 2021

**Authors:** Shaista Kanwal, Tahir Ghaffar, Azizul Hasan Aamir, Khalid Usman

**Affiliations:** 1 Dr. Shaista Kanwal, FCPS Medicine, MRCP (UK). Department of Diabetes, Endocrinology and Metabolic Diseases, Hayatabad Medical Complex, Peshawar, Pakistan; 2 Dr. Tahir Ghaffar, FCPS Endocrinology, FCPS Medicine, MRCP (UK). Department of Diabetes, Endocrinology and Metabolic Diseases, Hayatabad Medical Complex, Peshawar, Pakistan; 3 Dr. Azizul Hasan Aamir, MRCP (UK), FRCP, FACE. Department of Diabetes, Endocrinology and Metabolic Diseases, Hayatabad Medical Complex, Peshawar, Pakistan; 4 Dr. Khalid Usman, MRCP (UK), MRCP (IR). Department of Diabetes, Endocrinology and Metabolic Diseases, Hayatabad Medical Complex, Peshawar, Pakistan

**Keywords:** Type-2 Diabetes mellitus, Non-alcoholic fatty liver disease, HbA1c

## Abstract

**Objective:**

Non-Alcoholic Fatty Liver Disease (NAFLD) is emerging as a major public health problem globally especially in patients with Type-2 Diabetes mellitus (T2DM). This study aimed to assess the frequency of NAFLD in patients with T2DM and to study its associated risk factors.

**Methods:**

This descriptive study was conducted from April 2020 to October 2020 at the Hayatabad Medical Complex, Peshawar. Adult patients with T2DM were included in the study and underwent abdominal ultrasound for the identification of NAFLD. All the relevant clinical and biochemical characteristics were measured.

**Results:**

Out of 384 participants, 236 patients (61.5%) had NAFLD on ultrasound. Patients with NAFLD had higher mean BMI, higher HbA1c, increased waist circumference, raised ALT, higher triglyceride, and low HDL. Logistic regression analysis revealed a statistically significant association with central obesity (OR = 5.448, 95% CI = 1.416-20.959, p = 0.014), higher BMI (OR = 4.435, 95% CI = 2.127-9.246, p < 0.0001), higher HbA1c [> 11%] (OR = 3.602, 95% CI = 1.438-9.019, p = 0.006), and elevated ALT (OR = 3.211, 95% CI = 1.509-6.835, p = 0.002). The highest odds for NAFLD were found for hypertriglyceridemia (OR = 11.624, 95% CI = 5.405-24.998, p < 0.0001) and low HDL (OR = 11.543, 95% CI = 2.590-51.439, p = 0.001), respectively.

**Conclusions:**

High frequency of NAFLD along with its associated clinical and laboratory risk factors were revealed. This underpins the significance of screening T2DM patients for NAFLD and assessment for and modification of its associated risk factors in routine clinical practice.

## INTRODUCTION

Non-alcoholic fatty liver disease (NAFLD) comprises of a spectrum of liver diseases, ranging from fatty liver to non-alcoholic steatohepatitis (NASH) which is accompanied by inflammation.^1^ NAFLD is a well-known cause of Chronic liver disease (CLD), hepatocellular carcinoma and liver transplant, and is related with an increased risk of Type-2 Diabetes mellitus (T2DM), chronic kidney disease, cardiovascular disease and malignancies.^2^ Patients with NAFLD are found clinically to have elements of metabolic syndrome such as obesity, T2DM, hypertension and hyperlipidemia, among which T2DM is a crucial risk factor for the occurrence of NAFLD and a significant predictor of unfavorable clinical outcomes.^3^ The presence of insulin resistance and compensatory hyperinsulinemia can explain this association between NAFLD and T2DM.^4^

Global prevalence of NAFLD in the general public is around 15-30%, however the prevalence amongst patients with obesity or T2DM is 70-80%.^5^ This supports a bidirectional relationship between T2DM and NAFLD, suggesting that these metabolic disorders share a common pathogenic mechanism.^6^ Moreover, the concomitant presence of T2DM and NAFLD aggravates insulin resistance, favors the promotion of dyslipidemia and makes optimal glycemic control difficult to achieve, thereby developing major adverse cardiovascular events.^7^ Transabdominal ultrasound scan (US) is a widely used diagnostic modality for NAFLD due its free availability, high sensitivity, low cost and non-invasive nature compared to the gold standard histological assessment which is limited in use because of invasive nature and associated complications. Although, the progressing epidemic of obesity and T2DM is accompanied by the rising incidence of NAFLD, the factors implicated for this raised prevalence of NAFLD in diabetics is only poorly studied.^5^ There is a rising tendency of these factors among Asian population who are genetically more susceptible to the presence of insulin resistance due to the difference in the amount and distribution of body fat from Caucasians.^8^

However, in this regard very limited studies are performed in Pakistan where patients with T2DM are not screened routinely for NAFLD. In a hospital based study, the prevalence of NAFLD was 47%, whereas the frequency of NAFLD and NASH among 26 diabetic patients was 75% and 22.5% respectively.^9^ In a study by Taseer et al. the frequency of NAFLD among patients with T2DM was 51%.^10^ Another study reported 78.7% frequency of NAFLD in patients with T2DM.^11^ However, new data is required to appraise the different factors responsible for the occurrence of NAFLD among Type-2 diabetics. Therefore, the aim of this study was to determine the frequency of NAFLD in our Type-2 diabetic patients and to analyze the various clinical and laboratory parameters associated with the presence of NAFLD in patients with T2DM. This will not only provide valuable information about the burden of NAFLD among diabetics but will also determine its predictive risk factors, which will in turn strengthen the importance of primary prevention and prompt management.

## METHODS

This descriptive cross-sectional study was conducted from April 2020 to October 2020 at the Department of Diabetes, Endocrinology and Metabolic Diseases, Hayatabad Medical Complex, Peshawar, Pakistan. The study was approved by the ethical committee of the hospital under Ref. No. 276/HEC/B&PSC/2020 Dated 06/03/2020. The patients were selected through nonprobability consecutive sampling. Taking 51% proportion of NAFLD in patients with T2DM,^10^ 95% confidence interval and 5% margin of error, the calculated sample size was 384 using WHO calculator for sample size calculation.^12^ After getting written informed consent, adult patients with T2DM were enrolled in the study. Patients with type 1 DM, pregnancy, malignancy, history of any quantity of alcohol intake, history of intake of traditional medications, CLD, previous history of acute hepatitis of any cause including viral causes, known cases of autoimmune hepatitis, Wilson disease, hemochromatosis and alpha 1 antitrypsin deficiency were excluded from the study. Similarly, patients who had used methotrexate, corticosteroids, tamoxifen, amiodarone, low dose estrogen (≤ 0.3 mg conjugated estrogen) in the last one year and those on active drug therapy for obesity were also excluded from the study.

Anthropometric measurements were obtained, and body mass index (BMI) was determined. As per WHO guidelines for the Asian people; patients with a BMI ≤ 18.5 kg/m^2^ were labelled underweight, BMI between 18.5 to 22.9 kg/m^2^ as of normal weight, those with a BMI between 23 to 27.4 kg/m^2^ as overweight, whereas those with a BMI ≥ 27.5 kg/m^2^ were considered obese.^13^,^14^ Males with a waist circumference of more than 90 cm, and females with a waist circumference of more than 80 cm were labelled to have central obesity.^15^ Diabetes Mellitus (DM) was diagnosed according to the American Diabetes Association (ADA) diagnostic criteria.^16^ Hypertension was defined as per the American College of Cardiology/American Heart Association (ACC/AHA) 2017 guidelines.^17^ Laboratory factors like fasting plasma glucose (FBS), two hours postprandial plasma glucose (RBS), glycated hemoglobin (HbA1c), serum creatinine and uric acid, blood urea, fasting lipid profile, serum bilirubin, serum alanine aminotransferase (ALT) and alkaline phosphatase were evaluated. Serological markers of viral hepatitis (Hepatitis B and C) were also measured. Patients were considered to have dyslipidemia if they satisfied one of these criteria: Low density lipoprotein- cholesterol (LDL-C) > 100 mg/dl, total cholesterol > 200 mg/dl, triglycerides > 150 mg/dl, or High-density lipoprotein-cholesterol (HDL-C) < 40 mg/dl in males and < 50 mg/dl in females. Similarly, patients with triglycerides of > 150 mg/dl were considered to have hypertriglyceridemia.^15^ Using NHANES III criteria, males with ALT > 40 IU/L and females with ALT > 31 IU/L were considered to have elevated ALT.^18^

To identify fatty changes in the liver, all study participants underwent abdominal ultrasonography utilizing a high-resolution B-mode ultrasound system. To prevent interpersonal variation, all ultrasounds were performed by a single experienced radiologist. Increased hepatic echogenicity in comparison to spleen and kidney, with diminution of the wave, waning of demarcation of the diaphragm, and inadequate delineation of the intra hepatic framework were used to detect NAFLD on ultrasound. One or two of these criteria were required to be accomplished to prevent false positive results.^19^

### Statistical Analysis

Statistical analysis was accomplished by means of SPSS version 20. Quantitative data were analyzed for mean ± standard deviation, whereas percentages were calculated for categorical variables. Patients with and without NAFLD were compared. Categorical variables and quantitative variables between the two groups were compared by performing chi-square test and independent sample’s t-test, respectively. Various clinical and laboratory features associated with the occurrence of NAFLD were evaluated by performing multivariate logistic regression analysis. Each of independent variable was analyzed for odds ratio (ORs) along with its 95% confidence intervals (CIs). P-value of less than 0.05 was considered statistically significant.

## RESULTS

A total of 384 participants were enrolled for the study, of whom there were 166 (43.23%) males and 218 (56.77%) females. The mean age, BMI, waist circumference and mean duration of T2DM are presented in Table-I. Overall, NAFLD was present in 236 (61.5%) study participants. Moreover, patients having NAFLD were compared with patients having no ultrasonographic evidence of NAFLD. There was no statistically significant difference between the two groups regarding mean age and gender distribution. Though, there was a statistically significant difference amongst the two groups in terms of mean BMI, waist circumference, T2DM duration, systolic and diastolic BP, HbA1c, triglycerides, total cholesterol, LDL and HDL cholesterol, serum uric acid and ALT. The baseline demographic and laboratory characteristics along with the comparison of clinical and laboratory findings between the two groups of patients are presented in Table-I and Table-II, respectively.

**Table-I:** Comparison of baseline clinical characteristics of Type-2 DM patients with and without NAFLD.

Patient Characteristic		All Patients (n= 384)	Without NAFLD (n=148)	With NAFLD (n=236)	*p*-value
Age (years)		55.22 ± 7.75	54.41 ± 7.40	55.73 ± 7.93	0.1
Gender	Male	166 (43.2%)	70 (47.3%)	96 (40.7%)	0.2
	Female	218 (56.78%)	78 (52.7%)	140 (59.3%)	
BMI (Kg/m^2^)		27.72 ±4.7936	24.555 ±3.3012	29.708 ±4.5117	<0.0001
BMI Categories (Kg/m^2^)	< 18.5	4 (1.04%)	4 (2.7%)	0 (0%)	<0.0001
	18.5 -22.9	61 (15.9%)	45 (30.4%)	16 (6.8%)	
	23-27.4	133 (34.6%)	70 (47.3%)	63 (26.7%)	
	≥ 27.5	186 (48.4%)	29 ((19.6%)	157 (66.5%)	
Waist Circumference (cm)	105.76 ± 15.214	94.39 ± 11.012	112.89 ± 12.996	<0.0001
Central Obesity	No	44 (11.5%)	40 (27.02%)	4 (1.69%)	<0.0001
	Yes	340 (88.5%)	108 (72.97%)	232 (98.31%)	
Smoking	No	284 (73.96%)	128 (13.5%)	156 (33.9%)	<0.0001
	Yes	100 (26.04%)	20 (86.5%)	80 (66.1%)	
Duration of Type 2 DM (years)		12.87 ± 6.347	11.74 ± 6.089	13.58 ± 6.415	0.006
Duration of Type-2 DM categories (years)	< 5	35 (9.1%)	15 (10.1%)	20 (8.5%)	0.004
	5 – 10	61 (15.9%)	32 (21.6%)	29 (12.3%)	
	10 – 15	144 (37.5%)	61 (41.2%)	83 (35.2%)	
	> 15	144 (37.5%)	40 (27.03%)	104 (44.07%)	
Hypertension	No	137 (35.7%)	82 (55.4%)	55 (23.3%)	<0.0001
	Yes	247 (64.3%)	66 (44.6%)	181 (76.7%)	
Systolic BP (mmHg)		136.07 ± 17.742	129.19 ± 17.203	140.38 ± 16.716	<0.001
Diastolic BP (mmHg)		83.19 ± 9.724	79.19 ± 8.769	85.70 ± 9.467	<0.001

**Table-II:** Comparison of laboratory parameters of Type-2 DM patients with and without NAFLD.

Lab Parameter		All patients (n=384)	Without NAFLD (n=148)	With NAFLD (n=236)	*p*-value
FBS (mg/dl)		184.17 ± 73.311	161.53 ± 62.401	198.36 ±76.148	<0.0001
RBS (mg/dl)		260.35 ± 87.799	232.12 ± 75.703	278.05 ± 90.345	<0.0001
HbA1c (%)		11.345 ± 2.3153	10.134 ± 2.0455	12.105 ± 2.1483	<0.0001
HbA1c Categories (%)	7-8.9	75 (19.5%)	49 (33.1%)	26 (11.01%)	<0.0001
	9-10.9	88 (22.9%)	50 (33.8%)	38 (16.1%)	
	≥ 11	221 (57.6%)	49 (33.1%)	172 (72.9%)	
Total cholesterol (mg/dl)		174.11 ± 117.565	140.38 ± 32.807	195.26 ± 143.829	<0.0001
Triglycerides (mg/dl)		206.90 ± 100.638	134.20 ± 45.280	252.50 ± 99.031	<0.0001
LDL (mg/dl)		98.46 ± 34.627	80.01 ± 21.465	110.02 ± 36.296	<0.0001
HDL (mg/dl)		30.70 ± 10.479	36.06 ± 10.842	27.33 ± 8.714	<0.0001
Serum uric acid (mg/dl)		4.402 ± 1.5109	3.602 ± 1.3424	4.903 ± 1.3921	<0.0001
Alanine transaminase (ALT) (U/L)		31.26 ± 17.697	22.52 ± 13.613	36.74 ± 17.78	<0.0001
ALT categories	Normal	232 (60.4%)	121 (81.8%)	111 (47.0%)	<0.0001
	Elevated	152 (39.6%)	27 (18.2%)	125 (53.0%)	
Dyslipidemia	No	63 (16.4%)	57 (38.5%)	6 (2.5%)	<0.0001
	Yes	321 (83.6%)	91 (61.5%)	230 (97.5%)	
Low HDL	No	22 (5.7%)	18 (12.2%)	4 (1.7%)	<0.0001
	Yes	362 (94.3%)	130 (87.8%)	232 (98.3%)	
Hypertriglyceridemia	No	134 (34.9%)	110 (74.3%)	24 (10.2%)	<0.0001
	Yes	250 (65.1%)	38 (25.7%)	212 (89.8%)	

The results of the multivariate logistic regression analysis are shown in Table-III. These results showed that smoking, hypertension, central obesity, obesity, higher HbA1c (≥ 11%), elevated ALT, low HDL and hypertriglyceridemia were having independent association with the presence of NAFLD on ultrasound in T2DM patients. High triglyceride level and low HDL levels were the variables with the strongest association, conferring 11.6- and 11.5-fold increased likelihood of NAFLD in T2DM patients, respectively.

**Table-III:** Logistic Regression analysis for risk factors associated with NAFLD in patients with Type-2 DM

Variable		Odds Ratio (OR)	95% confidence interval	*p*-value
Gender	Male	1.0		
	Female	1.582	0.670-3.736	0.296
Smoking	No	1.0		
	Yes	5.232	1.784-15.340	0.003
Hypertension	No	1.0		
	Yes	2.221	1.111-4.438	0.024
Central obesity	No	1.0		
	Yes	5.448	1.416-20.959	0.014
BMI Categories (kg/m^2^)	< 27.5 (Non-Obese)	1.0		
	≥ 27.5 (Obese)	4.435	2.127-9.246	<0.0001
HbA1c categories (%)	7-8.9	1.0		
	9-10.9	1.616	0.587-4.455	0.35
	≥11	3.602	1.438-9.019	0.006
ALT	Normal	1.0		
	Elevated	3.211	1.509-6.835	0.002
HDL	Normal	1.0		
	Reduced	11.543	2.590-51.439	0.001
Triglyceride	Normal	1.0		
	Elevated	11.624	5.405-24.998	<0.0001
Dyslipidemia	No	1.0		
	Yes	1.773	0.580-5.426	0.315

## DISCUSSION

Around 61.5% of the study participants with T2DM satisfied the ultrasound criteria for NAFLD. This suggests a higher burden of NAFLD in patients with T2DM in our local population. The frequency of NAFLD in the South East Asian population ranges from 9% to 45%, and in patients with T2DM it varies from 6% to 62%.^20^ The 61.5% frequency of NAFLD in T2DM patients in the present study is very similar to that reported by a study conducted by Herath et al. in subjects having T2DM in Sri Lanka (61.9%).^5^

Patients who had NAFLD had higher values of mean systolic and diastolic blood pressure, mean FBS, mean RBS, LDL cholesterol, triglycerides and HbA1c compared to those without NAFLD. These were consistent with the findings demonstrated by Dai et al. and Butt et al. in their studies.^4^,^8^ Majority of the patients who had NAFLD were smokers and on logistic regression analysis, it was evident that smoking conferred an approximately five-fold higher risk of NAFLD. These findings were comparable to those by Agarwal et al. who demonstrated that patients in the NAFLD group had a higher frequency of hypertension and smoking.^21^

The mean BMI of patients in this study in the NAFLD group was 29.7 (± 4.5) kg/m^2^ compared to 24.5 (± 3.3) kg/m^2^ in the non-NAFLD group. A study conducted by Targher et al. in T2DM patients revealed that patients with NAFLD had a mean BMI of 28.3 kg/m^2^ compared to 26.5 kg/m^2^ in patients with no NAFLD. These results were closely related to the mean BMI in this study.^22^ This study also reported a very high mean waist circumference (112.89 ± 12.996) in the study participants having NAFLD compared to those with no evidence of NAFLD (94.39 ± 11.012). Similar associations with central obesity have been described in some earlier studies as well.^4^,^5^ Moreover, obesity (OR = 4.435, 95% CI = 2.127-9.246) and central obesity (OR = 5.448, 95% CI = 1.416-20.959) were independently associated with NAFLD. This was also observed in a study by Leite et al. who revealed that obesity and central obesity were independent predictive factors for NAFLD in the diabetic population.^23^

Studies have shown that elevated ALT could act as an independent component in the occurrence of metabolic complications.^24^ Overall, in the current study, 152 (39.6%) participants had elevated ALT and those having NAFLD had a higher proportion of participants with raised ALT compared to those without NAFLD (53.0% versus 18.2%, *p* < 0.0001). This is in contrast to the 16.4% frequency of elevated ALT in Type-2 diabetic patients with NAFLD in a study conducted by Butt et al.^8^ This difference in the proportion of raised ALT between this study and our study could be due to that patients included in our study had a relatively longer duration of T2DM, had greater mean BMI, higher mean waist circumference and higher mean HbA1c. Moreover, Butt et al. conducted their study on patients with newly diagnosed T2DM in comparison to this study where majority (44.07%) of subjects with NAFLD had T2DM for longer than 15 years. In addition, mean HbA1c in the NAFLD group in our study was 12.105 ± 2.1483 compared to their study where mean HbA1c was 8.13±1.69.^8^ Logistic regression analysis also demonstrated that raised ALT (OR = 3.211, 95% CI = 1.509-6.835) and HbA1c of ≥ 11% (OR = 3.602, 95% CI = 1.438-9.019) had independent associations with the development of NAFLD amongst T2DM patients. This finding is comparable to another study where ALT was associated significantly with the possibility of NAFLD.^25^ Likewise, a study by Ma et al. revealed the association of HbA1c with NAFLD.^26^

Another remarkable finding of our study was that 89.8% of the patients with NAFLD had hypertriglyceridemia, 88.1% of patients had both hypertriglyceridemia and central obesity and 98.3% of the patients were having reduced HDL. These results are comparable to the findings presented by Butt et al. in their study who reported that odds of NAFLD with both central obesity and high triglycerides was 3.7.^8^ A study by Radu et al. demonstrated that central obesity and hypertriglyceridemia had higher odds ratio for NAFLD, concluding that these factors were independent determinants of NAFLD.^27^

These findings are quite alarming as it is known that increased waist circumference along with high plasma triglyceride level are important cardiovascular risk factors and serves as strong determinants of cardiovascular disease.^28^ Our study revealed that central obesity, higher BMI, higher HbA1c, hypertriglyceridemia, low HDL levels, smoking and raised ALT were the elements having significant association with the presence of NAFLD. These results open new avenues for treatment in T2DM patients by focusing not only on glycemic control but also targeting these various cardiometabolic risk factors.

### Limitations of the study

Although this study exclusively studied Type-2 diabetic patients and various parameters are compared in detail between patients with and without NAFLD, it has few limitations. Firstly, confirmation with liver biopsy was not undertaken because that is invasive and was neither feasible nor cost effective in these asymptomatic patients. Secondly, majority of T2DM patients were on statins as part of multipronged approach to achieve metabolic control and it was unethical to stop their statins or exclude them just because they were on statins. Thirdly, this study was performed at a single center in a tertiary care hospital and generalization of these findings should be done once confirmed by large scale multicenter studies. Despite this, our study revealed that approximately two out of three patients with T2DM had NAFLD.

## CONCLUSION

This study reported an increased frequency of NAFLD in our diabetic population and evaluated in depth the risk factors associated with NAFLD, underpinning the significance of carrying further large-scale studies to assess the effects of lifestyle modification in the form of physical activity and dietary modifications on the status of NAFLD and glycemic control. Taking in to account the results of this study, patients and their treating physicians should emphasize on the modification of the associated factors and it is also advisable to screen diabetic patients for this condition in routine clinical practice. Early detection and timely management will help promote healthy lifestyle and prevent long term complications of the condition.

### Author’s Contribution:

**SK** conceived, designed, did literature review, performed statistical analysis & drafted the manuscript.

**TG** participated in analysis and interpretation of data, and helped in drafting the manuscript.

**AHA** conceived, designed, and critically revised the manuscript.

**KU** helped in analysis and interpretation of data, and critically revised the manuscript.

All authors provided final approval for publication of the manuscript, and are responsible for the integrity of the study.
